# Lifestyle factors and multimorbidity: a cross sectional study

**DOI:** 10.1186/1471-2458-14-686

**Published:** 2014-07-05

**Authors:** Martin Fortin, Jeannie Haggerty, José Almirall, Tarek Bouhali, Maxime Sasseville, Martin Lemieux

**Affiliations:** 1 Département de médecine de famille et de médecine d’urgence, Université de Sherbrooke, Québec, Canada; 2 Centre de santé et de services sociaux de Chicoutimi, 305 St-Vallier, Chicoutimi, Québec G7H 5H6, Canada; 3 Faculty of medicine, McGill University, Montreal, Canada

**Keywords:** Multimorbidity, Lifestyle factors, Smoking habit, Alcohol consumption, Fruit and vegetable consumption, Physical activity, Body mass index

## Abstract

**Background:**

Lifestyle factors have been associated mostly with individual chronic diseases. We investigated the relationship between lifestyle factors (individual and combined) and the co-occurrence of multiple chronic diseases.

**Methods:**

Cross-sectional analysis of results from the Program of Research on the Evolution of a Cohort Investigating Health System Effects (PRECISE) in Quebec, Canada. Subjects aged 45 years and older. A randomly-selected cohort in the general population recruited by telephone. Multimorbidity (3 or more chronic diseases) was measured by a simple count of self-reported chronic diseases from a list of 14. Five lifestyle factors (LFs) were evaluated: 1) smoking habit, 2) alcohol consumption, 3) fruit and vegetable consumption, 4) physical activity, and 5) body mass index (BMI). Each LF was given a score of 1 (unhealthy) if recommended behavioural targets were not achieved and 0 otherwise. The combined effect of unhealthy LFs (ULFs) was evaluated using the total sum of scores.

**Results:**

A total of 1,196 subjects were analyzed. Mean number of ULFs was 2.6 ± 1.1 SD. When ULFs were considered separately, there was an increased likelihood of multimorbidity with low or high BMI [Odd ratio (95% Confidence Interval): men, 1.96 (1.11-3.46); women, 2.57 (1.65-4.00)], and present or past smoker [men, 3.16 (1.74-5.73)]. When combined, in men, 4-5 ULFs increased the likelihood of multimorbidity [5.23 (1.70-16.1)]; in women, starting from a threshold of 2 ULFs [1.95 (1.05-3.62)], accumulating more ULFs progressively increased the likelihood of multimorbidity.

**Conclusions:**

The present study provides support to the association of lifestyle factors and multimorbidity.

## Background

Public health programs designed to fight chronic diseases should always consider lifestyle factors as important targets for prevention. The importance of lifestyle factors in health and disease has been the subject of many studies, and much of the information has been collected and analyzed in reports that unequivocally show a close relationship between lifestyle factors and many chronic diseases [1-4].

Because of its association with chronic diseases, each individual lifestyle factor becomes an important element to take into account in the prevention of these diseases. Furthermore, the combination of preventive lifestyle factors has also been reported to prevent the occurrence of chronic medical conditions [5-7]. Although the attributable risk of disease differs for individual lifestyle factors, epidemiologic evidence demonstrates that significant morbidity and mortality benefits are accumulated over any and all healthy lifestyle factors [7-11].

The link between lifestyle factors and chronic diseases has been studied mostly by analyzing individual lifestyle risk factors or their combinations associated with individual chronic diseases [11-18]. The association of lifestyle risk factors with the co-occurrence of multiple long-term or chronic diseases in one individual (multimorbidity) has been explored for physical activity in elderly patients [19], obesity [20-24], smoking [24], alcohol consumption [24], and nutrition [24,25]. However, the association of accumulating risk factors in the same individual and multimorbidity has not been explored. The aim of the present study was to analyze the association between individual lifestyle factors and their combinations with the occurrence of multimorbidity as well as the effect of their accumulation in an individual. We wanted to test the hypothesis that the accumulation of unhealthy lifestyle factors is associated with increased likelihood of multimorbidity

## Methods

### Study design and setting

The present study is a cross-sectional analysis conducted among a cohort of the general population recruited for a larger project (the Program of Research on the Evolution of a Cohort Investigating Health System Effects, PRECISE) [26] within the geographic boundaries of four local healthcare networks in Quebec, Canada. These networks are located in metropolitan, urban, rural and remote settings.

### Participants

A randomly-selected cohort was recruited from March to April 2010 through a telephone survey. The survey firm hired for this task generated a random list of telephone numbers mapped to the postal code areas that correspond to the administrative boundaries of the four networks identified. Once contact was made, staff selected the adult in the household with the most recent birthday to ensure random selection. Participants had to be aged between 25 and 75 years, able to respond to written and oral questions in English or French and reside in one of the four networks identified. The purpose was to include 2000 subjects in the study. For this reason, an initial over-recruit by 20% (2,400) was made.

### Data collection

At recruitment, participants reported on socio-demographic information (age, gender, family income, education, etc.). Two weeks after recruitment, participants completed a self-administered questionnaire (paper, internet or telephone response) that included the instruments to measure illness burden and lifestyle factors.

To capture the multi-dimensional nature of socio-economic status, we produced a data-driven classification of socio-economic status based on several variables that were considered important. The variables used were: education level, perceived financial situation, house ownership, presence or absence of medical insurance, and the possession of a retirement plan. Using a split sample, principal component analysis (PCA) was performed to summarize the different variables into smaller groups of “factors” (weighted sum of variable values). These factors then served as a basis for the classification of participants into four relatively homogenous socio-economic clusters. Definitional criteria for the four socio-economic clusters were:

1) Elite Group: College or university education AND perceived financial situation as “comfortable” or “very comfortable”.

2) Middle-high: College or university education OR perceived financial situation as “comfortable” or “very comfortable”.

3) Middle-low: High school education and perceived financial situation as “modestly comfortable” OR less than high school education and perceived financial situation less than “tight” but possess a retirement plan and complimentary medical insurance.

4) Low: Less than high school education and perceived financial situation less than “tight” but does not possess a retirement plan or complimentary medical insurance.

The outcome of interest, multimorbidity, was defined as the presence of three or more chronic conditions, measured by a simple count of the number of chronic conditions present in the subjects from a list of 14 conditions (hypertension, cholesterol elevated, asthma, chronic obstructive pulmonary disease, diabetes, thyroid disorder, osteoarthritis, rheumatoid arthritis, osteoporosis, colon problem, angina/coronary artery disease, stroke, congestive heart failure, and cancer) of the Disease Burden Morbidity Assessment by self-report [27,28]. The presence and intensity of lifestyle factors were evaluated using lifestyle-specific subscales from the Behavioural Risk Factor Surveillance System Questionnaire [29].

Lifestyle factors considered were: 1) smoking habit, 2) alcohol consumption, 3) fruit and vegetable consumption, 4) physical activity, and 5) body mass index (BMI). Each lifestyle factor was given a score of 1 (unhealthy) if recommended behavioural targets were not achieved and 0 otherwise. The criteria for classifying participants as not achieving recommended behavioural targets were the following: 1) present or past smoker; 2) high risk alcohol consumption (more than 10 standard drinks per week for women; more than 15 standard drinks per week for men) [30]; 3) insufficient fruit and vegetable consumption (less than five portions daily; 1 portion = 4 ounces or 125 ml); 4) not physically active (less than 20-30 min. of exercise 4 times per week); 5) low or high BMI (normal BMI: 18.5-24.9 Kg/m^2^) [31]. The combined effect of unhealthy lifestyle factors was evaluated using the total sum of lifestyle specific scores in each subject [7,13,32], yielding an overall score for combined unhealthy lifestyle factors ranging from 0 to 5.

### Data analysis

For this study, only patients of age 45 and over were considered in the analysis. The specific prevalence of multimorbidity was estimated by the number of unhealthy lifestyle factors and sex. Calculations of relative risks and 95% confidence intervals (95% CI) were used to evaluate differences in the prevalence of multimorbidity in the groups with different numbers of lifestyle factors, using the prevalence of multimorbidity in the group with lower unhealthy lifestyle factors as the reference category.

We used logistic regression modeling to calculate the odd ratios for the likelihood of multimorbidity by lifestyle factor, adjusting for age, education, and socioeconomic class. All these covariates have been reported to be associated with multimorbidity.

### Ethical aspects

Major ethical concerns were ensuring confidentiality and maintaining participation throughout the study period. Only the project coordinator and principal investigator had access to the link between nominal information and the unique study identification code. Subjects signed an informed consent to participate in the study, and the individual’s right to withdraw partially or completely was reiterated at data collection efforts. The study was approved by the scientific and ethics committees of the Centre de santé et de services sociaux de Chicoutimi, as well as the Research Ethics Committee of Hôpital Charles Lemoyne, Quebec.

## Results

A total of 2,458 subjects were recruited. Among them, 2,409 subjects were eligible. A total of 1,718 subjects (70% of those initially recruited) returned the completed questionnaires. We included in the study 1,196 subjects of age 45 or older found in the sample. Characteristics of participants are shown in Table 1. The percentage of subjects not achieving recommended behavioural targets for the five lifestyle factors by sex are shown in Table 2. With the exception of physical activity, the percentage of subjects not achieving recommended behavioural targets for each lifestyle factor was statistically significant different between men and women. Due to the difference in distribution of adherence to lifestyle factors by gender, all the analyses were conducted separately for men and women.

**Table 1:** Characteristics of participants

	**Men (n = 515)**	**Women (n = 681)**	**p**
**Age: mean (SD)**	58.0 (7.9)	57.6 (8.2)	0.300
**Patients by number of diseases: n (%)**			0.508
0 disease	153 (29.7)	175 (25.7)	
1 disease	121 (23.5)	148 (21.7)	
2 diseases	102 (19.8)	154 (22.6)	
3 diseases	81 (15.7)	114 (16.7)	
4 diseases	31 (6.0)	48 (7.0)	
5 or more diseases	27 (5.2)	42 (6.2)	
**Socio-economic classes: n (%)**			0.399
Elite group	109 (21.2)	123 (18.1)	
Middle-high	206 (40.0)	296 (43.5)	
Middle-low	110 (21.4)	133 (19.5)	
Low	70 (13.6)	99 (14.5)	
Missing data	20 (3.9)	30 (4.4)	
**Education: n (%)**			0.220
Incomplete secondary school or lower	144 (28.0)	166 (24.4)	
Completed secondary school	162 (31.5)	209 (30.7)	
College	79 (15.3)	133 (19.5)	
University	129 (25.0)	170 (25.0)	
Missing data	1 (0.2)	3 (0.4)	

**Table 2:** Percentage of subjects not achieving recommended behavioural targets for each lifestyle factor

**Lifestyle factor**	**Men**	**Women**	**p**
**Smoking habit:** Present or past smoker	70.7	58.9	0.000
**Fruit and vegetable consumption:** Less than five portions* daily	62.3	37.6	0.000
**Physical activity:** Less than 20–30 min. 4 times/week	72.8	71.2	0.641
**Alcohol consumption:** High-risk drinking**	8.2	5.0	0.045
**Body Mass Index (BMI):** Low or high BMI***	71.3	57.3	0.000

* One portion = 4 ounces or 125 ml.

** High-risk drinking: More than 10 standard drinks per week for women; More than 15 standard drinks per week for men (1 standard drink = 1 small beer or 1 glass of wine or 1 glass of spirits/liquor).

*** Normal BMI: 18.5 – 24.9 Kg/m^2^.

**Figure 1:**
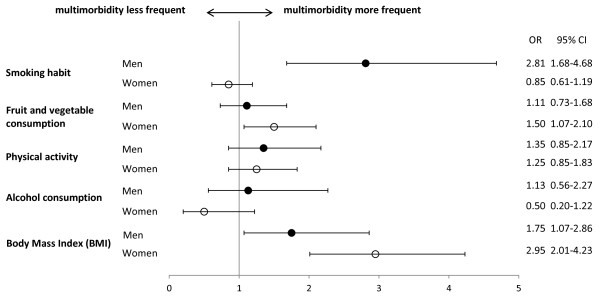
**Forest plot showing the bivariate effect of each unhealthy lifestyle factor on the likelihood of multimorbidity (3 or more chronic conditions).** Bars represent 95% confidence intervals (95% CI).

The odd ratios of the bivariate effect of each lifestyle factor for the presence of multimorbidity are shown in Figure 1. Physical activity and alcohol consumption showed no association with the presence of multimorbidity for either men or women. The odd ratios of the additional independent effect of each lifestyle factor over and above the others on the presence of multimorbidity, adjusted for age, education and socioeconomic class, are shown in Table 3. A lower or higher than normal BMI was the only lifestyle factor associated with higher likelihood of multimorbidity in both sexes. Being a current or past smoker was also associated with a higher likelihood of multimorbidity in men but not in women. No association with the presence of multimorbidity was found with not eating a recommended amount of fruit and vegetables, physical activity or alcohol consumption for either men or women.

**Table 3:** Odds ratios and 95% confidence intervals (95% CI) of the additional independent effect of each lifestyle factor over and above the others on the likelihood of multimorbidity (3 or more chronic conditions), adjusted for age, socioeconomic class, and education (p = 0.005 for interaction with sex in a model with men and women together)

	**Men**			**Women**		
**Lifestyle factors**	**p**	**Odds ratio**	**95% CI**	**p**	**Odds ratio**	**95% CI**
**Smoking habit:** Present or past smoker	0.000	3.16	1.74-5.73	0.907	0.98	0.64-1.49
**Fruit and vegetable consumption:** Less than five portions* daily	0.838	0.95	0.59-1.54	0.089	1.46	0.94-2.26
**Physical activity:** Less than 20–30 min. 4 times/week	0.257	1.38	0.79-2.42	0.290	1.29	0.81-2.06
**Alcohol consumption:** High-risk drinking**	0.872	0.94	0.43-2.03	0.709	0.83	0.31-2.23
**Body Mass Index (BMI):** Low or high BMI***	0.020	1.96	1.11-3.46	0.000	2.57	1.65-4.00
**Covariates**						
**Age**	0.000	1.07	1.04-1.11	0.000	1.11	1.08-1.15
**Socioeconomic class**						
Elite group	-			-		
Middle-high	0.751	0.90	0.46-1.76	0.331	1.35	0.74-2.46
Middle-low	0.900	0.94	0.38-2.37	0.257	1.64	0.70-3.87
Low	0.508	1.40	0.52-3.80	0.064	2.49	0.95-6.54
**Education**						
Incomplete secondary school or lower	-			-		
Completed secondary school	0.643	1.16	0.62-2.16	0.406	1.31	0.69-2.45
College	0.832	1.10	0.44-2.75	0.821	1.11	0.45-2.74
University	0.210	0.58	0.25-1.36	0.647	0.82	0.35-1.93

* One portion = 4 ounces or 125 ml.

** High-risk drinking: More than 10 standard drinks per week for women; More than 15 standard drinks per week for men (1 standard drink = 1 small beer or 1 glass of wine or 1 glass of spirits/liquor).

*** Normal BMI: 18.5 – 24.9 Kg/m^2^.

**Figure 2:**
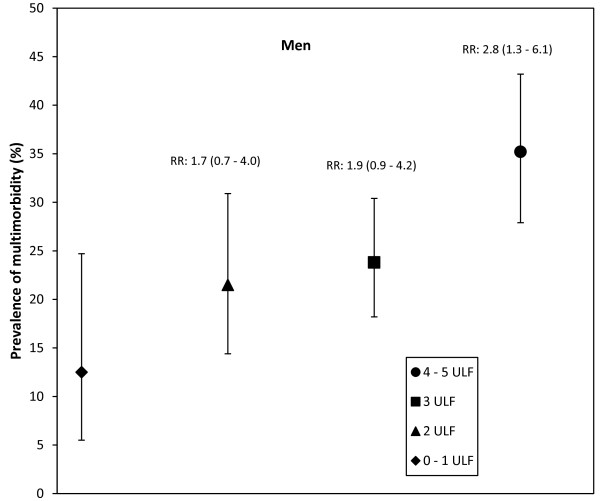
**Prevalence of multimorbidity and number of unhealthy lifestyle factors (ULF) in men.** Bars represent 95% confidence intervals (95% CI). Relative risks (RR) and 95% CI for each group, compared with the 0–1 LF group, are also shown.

**Figure 3:**
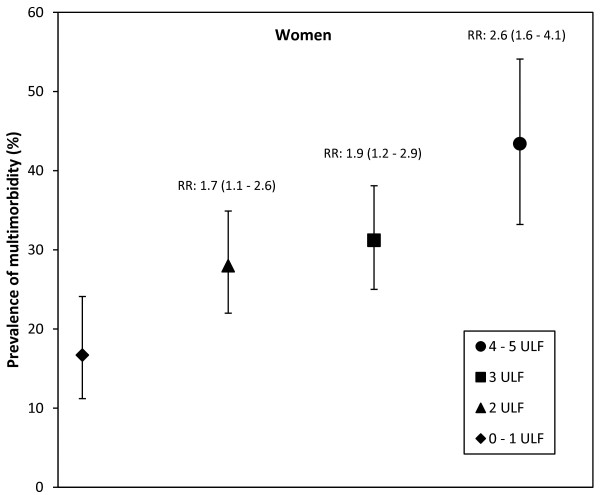
**Prevalence of multimorbidity and number of unhealthy lifestyle factors (ULF) in women.** Bars represent 95% confidence intervals (95% CI). Relative risks (RR) and 95% CI for each group, compared with the 0–1 LF group, are also shown.

The overall mean number ± SD of unhealthy lifestyle factors in the cohort was 2.6 ± 1.10. The percentage of subjects with 0, 1, 2, 3, 4, and 5 unhealthy lifestyle factors by sex are shown in Table 4. This analysis was limited to those subjects whose total lifestyle score could be calculated. Subjects with one or more missing values were not included in the analysis. Because the number of subjects who had 0 or 5 unhealthy lifestyle factors was small, these categories were combined as a single group with the categories of 1 and 4 unhealthy lifestyle factors, respectively. Therefore, we analyzed the categories of 0-1, 2, 3, and 4-5 unhealthy lifestyle factors. Results of the logistic regression analysis for the likelihood of multimorbidity associated with the number of unhealthy lifestyles factors adjusted for age, education and socioeconomic class are shown in Table 5. The presence of 2 unhealthy lifestyle factors, or more, significantly increased the likelihood of multimorbidity in women; accumulating unhealthy lifestyle factors progressively increased the likelihood of multimorbidity. In men, only the presence of 4-5 unhealthy lifestyle factors significantly increased the likelihood of multimorbidity.Prevalence of multimorbidity by sex and number of unhealthy lifestyle factors as well as relative risks are shown in Figures 2 and 3. Relative risks above one in the 95% confidence intervals were observed with 2, 3, and 4-5 unhealthy lifestyle factors in women. Relative risks above one in the 95% confidence intervals were observed only with 4-5 unhealthy lifestyle factors in men.

**Table 4:** Subjects with 0, 1, 2, 3, 4, and 5 unhealthy lifestyle factors (men, n = 471; women, n = 580)

**Number of unhealthy lifestyle factors**	**Men n (%)**	**Women n (%)**
**0**	10 (2.1)	33 (5.7)
**1**	38 (8.1)	93 (16.0)
**2**	93 (19.7)	182 (31.4)
**3**	185 (39.3)	189 (32.6)
**4**	135 (28.7)	79 (13.6)
**5**	10 (2.1)	4 (0.7)

**Table 5:** Odds ratios for the likelihood of multimorbidity (3 or more chronic conditions) by number of unhealthy lifestyle factors (ULF), adjusted for age, socioeconomic class, and education (p = 0.003 for interaction with sex in a model with men and women together)

	**Men**			**Women**		
**Number of ULFs**	**p**	**Odds ratio**	95% CI*	**p**	**Odds ratio**	**95% CI**
0-1	-	1		-	1	
2	0.178	2.24	0.69-7.21	0.036	1.95	1.05-3.62
3	0.058	2.93	0.96-8.94	0.003	2.53	1.36-4.71
4-5	0.004	5.23	1.70-16.1	0.001	3.39	1.65-6.95
**Covariates**						
**Age**	0.000	1.07	1.04-1.10	0.000	1.12	1.09-1.15
**Socioeconomic class**						
Elite group	-			-		
Middle-high	0.750	0.90	0.47-1.73	0.269	1.40	0.77-2.55
Middle-low	0.922	0.96	0.39-2.36	0.173	1.81	0.77-4.24
Low	0.565	1.34	0.50-3.58	0.041	2.74	1.04-7.20
**Education**						
Incomplete secondary school or lower	-			-		
Completed secondary school	0.691	1.13	0.61-2.10	0.410	1.31	0.69-2.49
College	0.860	1.08	0.44-2.67	0.740	1.16	0.48-2.85
University	0.279	0.63	0.28-1.45	0.642	0.81	0.35-1.91

*CI = Confidence interval.

## Discussion

There is an important body of research showing that common lifestyle factors, either individually or combined, are associated with individual chronic conditions [1-7,11,14,18]. The present study suggests that some individual lifestyle factors as well as the combined effect of lifestyle factors are associated with the likelihood of the simultaneous presence of three or more chronic conditions in the same subject (multimorbidity). Considering lifestyle factors individually, the BMI and the smoking habit were lifestyle factors associated with a likelihood of multimorbidity. When lifestyle factors were combined, starting from a threshold of 2 lifestyle factors in women and 4–5 in men, accumulating unhealthy lifestyle factors progressively increased the likelihood of multimorbidity.

The lack of association with physical activity found in this study is at variance with a recent study reporting an inverse association between physical activity and multimorbidity among men aged 65–94 years [19]. Probably, the difference in age of subjects included in each study and different definitions of multimorbidity (2 or more vs. 3 or more chronic conditions) are at the origin of the discrepant results.

A recent publication reported that greater consumption of fruits and vegetable and whole grain products appear to lower the risk of multimorbidity [25]. These results are at variance with the finding in the present study that not eating a recommended amount of fruit and vegetables was not associated with a higher likelihood of multimorbidity. However, our results are in line with a study of Nadel and colleagues [24] in which adjustment for fruit and vegetable intake, physical activity, and alcohol consumption did not substantially influenced the associations between multimorbidity and education in both men and women. However, the results of Nadel and colleagues are at variance with our finding of an association of smoking habit and multimorbidity in men.

The BMI has been repeatedly reported to be associated with multimorbidity [20-24]. Our study has confirmed this association. Indeed, the BMI was the only lifestyle factor associated with a likelihood of multimorbidity in both sexes.

In the present study, accumulation of unhealthy lifestyle habits was associated with a higher likelihood of multimorbidity. These findings concur with previous reports about a significant association of morbidity and mortality benefits with the accumulation of healthy lifestyle habits [7-11].

The results of this study supporting the link between lifestyle factors and multimorbidity point to the hypothesis that preventive measures could be considered an intervention in the fight against multimorbidity. However, it should be emphasized that although the identification of risk factors is an important aspect in the search for prevention and intervention strategies to address the increasing problem of multimorbidity, so is the person-centered approach. The high prevalence of multimorbidity implies the need for a holistic approach beyond the management of individual diseases. A person-centered approach promoting healthy lifestyles would maximize the number of healthy lifestyles in each individual, starting with whichever behaviour the person is most ready to adopt. The health care provider can use or refer the patient to strength-building approaches, such as motivational interviewing, that support patients in their decision to adopt a healthy behaviour and then to build or maintain the motivation to meet their goals. Building on the self-efficacy achieved for one behaviour, other behaviours can then be targeted [33,34].

There are several limitations in our study. The cross-sectional design of the study does not allow us to make a causal inference, thus, results need to be interpreted with that in mind. The self-report of lifestyle data may result in some misclassification. Nevertheless, this type of misclassification would likely be random. Also, the lifestyle factors were dichotomized, and we lost information on any dose–response because there are graded associations between some of the lifestyle factors and the occurrence of chronic conditions. The crude dichotomous categorization of lifestyle factors might underestimate the true effect of the various risk factors. However, previous studies have demonstrated that dichotomized lifestyle factors have been useful for studying the association of lifestyle factors and the risk of cancer [7], the prevention of chronic diseases [13], and all-cause mortality [32] in large populations. Overall lifestyle scores similar to the one we developed for the present study were used in these previous studies [7,32]. Regarding the measurement of multimorbidity, we considered 14 frequent chronic conditions, and this has an influence on prevalence estimates of multimorbidity. We have previously reported that a longer list of conditions would result in higher prevalence estimates [35]. Also, we had to rely on the self-reported presence of chronic conditions to measure multimorbidity and, hence, either over-reporting or under-reporting may have occurred. Finally, the results of this study are generalizable to the Canadian population where the study was conducted. Additional studies will be needed to evaluate the association of lifestyle behaviours and multimorbidity in other locations or populations.

## Conclusions

This study provides support for the association of unhealthy lifestyle factors and multimorbidity. The increase in the likelihood of multimorbidity with the combined effect of unhealthy lifestyle factors may be used to hypothesise that the promotion of health positive lifestyle factors could be an intervention in the fight against multimorbidity.

## Competing interests

The authors declare that they have no competing interests.

## Authors’ contributions

MF and JH conceived the study and participated in its design and coordination. JA and TB conducted the analysis of data and drafted the manuscript. JH, MS and ML also contributed to the analysis and interpretation of data. MS and ML along with MF, JH, TB and JA revised the paper for important intellectual content. All authors gave the final approval of the version submitted. MF takes responsibility for the integrity of the work as a whole.

## Pre-publication history

The pre-publication history for this paper can be accessed here:

http://www.biomedcentral.com/1471-2458/14/686/prepub

## References

[B1] Policy and action for cancer prevention. Food, nutrition, and physical activity: a global perspective [http://www.dietandcancerreport.org/cancer_resource_center/downloads/Policy_Report.pdf]

[B2] World Health Report 2002: reducing risks, promoting healthy life [http://www.who.int/whr/2002/en/Overview_E.pdf] 10.1080/1357628031000116808 14741909

[B3] U.S. Department of Health and Human Services Physical Activity and Health: A Report of the Surgeon General 1996 Atlanta, GA: Department of Health and Human Services, Centers for Disease Control and Prevention, National Center for Chronic Disease Prevention and Health Promotion

[B4] U.S. Department of Health and Human Services The Health Consequences of Smoking: A Report of the Surgeon General 2004 Atlanta, GA: U.S. Department of Health and Human Services, Centers for Disease Control and Prevention, National Center for Chronic Disease Prevention and Health Promotion, Office on Smoking and Health

[B5] Cuenca-Garcia M Ortega FB Ruiz JR Gonzalez-Gross M Labayen I Jago R Martinez-Gomez D Dallongeville J Bel-Serrat S Marcos A Manios Y Breidenassel C Widhalm K Gottrand F Ferrari M Kafatos A Molnár D Moreno LA De Henauw S Castillo MJ Sjöström M HELENA Study Group Combined influence of healthy diet and active lifestyle on cardiovascular disease risk factors in adolescents Scand J Med Sci Sports 2014 24 553 562 10.1111/sms.12022 23237548

[B6] Akesson A Weismayer C Newby PK Wolk A Combined effect of low-risk dietary and lifestyle behaviors in primary prevention of myocardial infarction in women Arch Intern Med 2007 167 2122 2127 10.1001/archinte.167.19.2122 17954808

[B7] Sasazuki S Inoue M Iwasaki M Sawada N Shimazu T Yamaji T Tsugane S Combined impact of five lifestyle factors and subsequent risk of cancer: the Japan Public Health Center Study Prev Med 2012 54 112 116 10.1016/j.ypmed.2011.11.003 22155160

[B8] Breslow L Enstrom JE Persistence of health habits and their relationship to mortality Prev Med 1980 9 4 469 483 10.1016/0091-7435(80)90042-0 7403016

[B9] King DE Mainous AG 3rd Matheson EM Everett CJ Impact of healthy lifestyle on mortality in people with normal blood pressure, LDL cholesterol, and C-reactive protein Eur J Prev Cardiol 2013 20 1 73 79 10.1177/1741826711425776 21965516

[B10] Matheson EM King DE Everett CJ Healthy lifestyle habits and mortality in overweight and obese individuals J Am Board Fam Med 2012 25 1 9 15 10.3122/jabfm.2012.01.110164 22218619

[B11] Odegaard AO Koh WP Gross MD Yuan JM Pereira MA Combined lifestyle factors and cardiovascular disease mortality in Chinese men and women: the Singapore Chinese health study Circulation 2011 124 2847 2854 10.1161/CIRCULATIONAHA.111.048843 22104554 PMC3400937

[B12] Chiuve SE McCullough ML Sacks FM Rimm EB Healthy lifestyle factors in the primary prevention of coronary heart disease among men: benefits among users and nonusers of lipid-lowering and antihypertensive medications Circulation 2006 114 160 167 10.1161/CIRCULATIONAHA.106.621417 16818808

[B13] Ford ES Bergmann MM Kroger J Schienkiewitz A Weikert C Boeing H Healthy living is the best revenge: findings from the European Prospective Investigation Into Cancer and Nutrition-Potsdam study Arch Intern Med 2009 169 1355 1362 19667296 10.1001/archinternmed.2009.237

[B14] Forman JP Stampfer MJ Curhan GC Diet and lifestyle risk factors associated with incident hypertension in women JAMA 2009 302 401 411 10.1001/jama.2009.1060 19622819 PMC2803081

[B15] Kurth T Moore SC Gaziano JM Kase CS Stampfer MJ Berger K Buring JE Healthy lifestyle and the risk of stroke in women Arch Intern Med 2006 166 13 1403 1409 10.1001/archinte.166.13.1403 16832006

[B16] Mozaffarian D Kamineni A Carnethon M Djousse L Mukamal KJ Siscovick D Lifestyle risk factors and new-onset diabetes mellitus in older adults: the cardiovascular health study Arch Intern Med 2009 169 8 798 807 10.1001/archinternmed.2009.21 19398692 PMC2828342

[B17] Myint PK Luben RN Wareham NJ Bingham SA Khaw KT Combined effect of health behaviours and risk of first ever stroke in 20,040 men and women over 11 years’ follow-up in Norfolk cohort of European Prospective Investigation of Cancer (EPIC Norfolk): prospective population study BMJ 2009 338 b349 10.1136/bmj.b349 19228771 PMC2645849

[B18] Reis JP Loria CM Sorlie PD Park Y Hollenbeck A Schatzkin A Lifestyle factors and risk for new-onset diabetes: a population-based cohort study Ann Intern Med 2011 155 292 299 10.7326/0003-4819-155-5-201109060-00006 21893622 PMC3491359

[B19] Autenrieth CS Kirchberger I Heier M Zimmermann AK Peters A Doring A Thorand B Physical activity is inversely associated with multimorbidity in elderly men: results from the KORA-Age Augsburg study Prev Med 2013 57 17 19 10.1016/j.ypmed.2013.02.014 23485795

[B20] Agborsangaya CB Lau D Lahtinen M Cooke T Johnson JA Multimorbidity prevalence and patterns across socioeconomic determinants: a cross-sectional survey BMC Public Health 2012 12 201 10.1186/1471-2458-12-201 22429338 PMC3353224

[B21] Booth HP Prevost AT Gulliford MC Impact of body mass index on prevalence of multimorbidity in primary care: cohort study Fam Pract 2014 31 1 38 43 10.1093/fampra/cmt061 24132593 PMC3902211

[B22] de S Santos Machado V Valadares AL Costa-Paiva LH Osis MJ Sousa MH Pinto-Neto AM Aging, obesity, and multimorbidity in women 50 years or older: a population-based study Menopause 2013 20 8 818 824 10.1097/GME.0b013e31827fdd8c 23549445

[B23] De Souza Santos Machado V Valadares AL Da Costa-Paiva LS Moraes SS Pinto-Neto AM Multimorbidity and associated factors in Brazilian women aged 40 to 65 years: a population-based study Menopause 2012 19 5 569 575 10.1097/gme.0b013e3182455963 22415564

[B24] Nagel G Peter R Braig S Hermann S Rohrmann S Linseisen J The impact of education on risk factors and the occurrence of multimorbidity in the EPIC-Heidelberg cohort BMC Public Health 2008 8 384 10.1186/1471-2458-8-384 19014444 PMC2614432

[B25] Ruel G Shi Z Zhen S Zuo H Kroger E Sirois C Levesque JF Taylor AW Association between nutrition and the evolution of multimorbidity: the importance of fruits and vegetables and whole grain products Clin Nutr 2013 http://dx.doi.org/10.1016/j.clnu.2013.07.009 10.1016/j.clnu.2013.07.009 23931982

[B26] Haggerty J Fortin M Beaulieu M-D Hudon C Loignon C Préville M Roberge D At the interface of community and healthcare systems: a longitudinal cohort study on evolving health and the impact of primary healthcare from the patient’s perspective BMC Health Serv Res 2010 10 258 10.1186/1472-6963-10-258 20815880 PMC2940881

[B27] Bayliss EA Ellis JL Steiner JF Seniors’ self-reported multimorbidity captured biopsychosocial factors not incorporated into two other data-based morbidity measures J Clin Epidemiol 2009 62 550 557 10.1016/j.jclinepi.2008.05.002 18757178 PMC2743235

[B28] Poitras ME Fortin M Hudon C Haggerty J Almirall J Validation of the disease burden morbidity assessment by self-report in a French-speaking population BMC Health Serv Res 2012 12 35 10.1186/1472-6963-12-35 22333434 PMC3305524

[B29] Behavioral risk factor surveillance system. Questionnaires [http://www.cdc.gov/brfss/questionnaires.htm]

[B30] Alcohol and health in Canada: a summary of evidence and guidelines for low risk drinking [http://www.ccsa.ca/Eng/topics/alcohol/drinking-guidelines/Pages/default.aspx]

[B31] Indice de masse corporelle, selon le sexe [http://www.msss.gouv.qc.ca/statistiques/sante-bien-etre/index.php?Indice-de-masse-corporelle-selon-le-sexe]

[B32] Tamakoshi A Tamakoshi K Lin Y Yagyu K Kikuchi S Healthy lifestyle and preventable death: findings from the Japan Collaborative Cohort (JACC) Study Prev Med 2009 48 5 486 492 10.1016/j.ypmed.2009.02.017 19254743

[B33] King TK Marcus BH Pinto BM Emmons KM Abrams DB Cognitive-behavioral mediators of changing multiple behaviors: smoking and a sedentary lifestyle Prev Med 1996 25 6 684 691 10.1006/pmed.1996.0107 8936570

[B34] Sallit J Ciccazzo M Dixon Z A cognitive-behavioral weight control program improves eating and smoking behaviors in weight-concerned female smokers J Am Diet Assoc 2009 109 8 1398 1405 10.1016/j.jada.2009.05.009 19631046

[B35] Fortin M Hudon C Haggerty J van den Akker M Almirall J Prevalence estimates of multimorbidity: a comparative study of two sources BMC Health Serv Res 2010 10 111 10.1186/1472-6963-10-111 20459621 PMC2907759

